# Progress in Dermatology

**Published:** 1903-10-03

**Authors:** 


### PROGRESS IN DERMATOLOGY.

Howard[1] has examined the pathological condition in two cases of herpes, and finds that while herpes of the trunk is associated with hæmorrhage in the posterior spinal ganglia, that affecting the upper lip and nose was associated with hæmorrhage in the Gasserian ganglion: this he claims as a new observation. Wys, Sattler, Head and Carpenter report cases of hæmorrhage in the Gasserian ganglion associated with ophthalmic herpes, and the latter two authors describe changes in the Gasserian ganglion associated with inferior maxillary herpes. The author accordingly would hold that herpes of the lip is pathologically one form of herpes zoster, and divides the forms into (*a*) spontaneous or primary herpes, thought by Head and Carpenter, etc., to be a specific infectious disease, the specific causal agent of which has a special affinity for certain sensory ganglia (posterior spinal and Gasserian); (*b*) toxic herpes, dependent upon such poisons as arsenic or carbon mon-oxide; (*c*) that occurring with certain acute infectious diseases as pneumonia, cerebro-spinal meningitis and probably malarial and typhoid fevers. The lesions of ganglia and skin in all these are the same, and the processes therefore presumably are identical; (*d*) herpes simplex affecting lips and nose in coryza, gastro-intestinal intoxication, and affecting genitals. These have not been sufficiently investigated to be classified. No evidence exists for or against its connection with changes in the nervous system.

Another instance of an acute bullous eruption developing in a person whose occupation has brought him into connection with recently-killed animals, is described by Morley and Ransome.[2] Cases were collected previously by Pernet and Bullock, and in most of these a small septic wound or whitlow acted as the starting point of the disease. In the case cited, a butcher aet. 26, there was also a septic wound on the left middle finger, with a circinate scaly patch on the back of the left forearm. This latter was treated with pure formalin and dressed with white precipitate ointment, the finger being also dressed with the ointment. A slight dermatitis ensued on the treatment of the scaly patch, which extended gradually over the body, leading to a general erythema with a bullous eruption, the blebs being small. The patient ultimately recovered. The authors believe that the eruption was not due to the formalin, but was in direct association with the septic wound of the finger. Whitfield[3] brings forward notes on two cases of acute pemphigus in infants and discusses the bacteriological ætiology. In the culture from one of these cases and in another previous case he has found streptococci. In the second of the reported cases no culture was made, but it was a very general case and microscopic sections showed large masses of cocci which stained well by Gram's method. Pemphigus neonatorum is now accepted as an impetiginous condition, and Whitfield asks whether these cases of acute pemphigus in infants may not be due to the same organism. In the second of his cases death took place about five hours after admission, although the child seemed to be remarkably little disturbed by the disease previously. The writer discusses the entity of Ritter's dermatitis exfoliativa neonatorum, the exact description of which tallies with his second reported case. As this was due to pus cocci he raises in question the existence as a separate disorder of Ritter's disease.

In dealing with eruptions produced by drugs, Pernet[4] remarks that in attempting to arrive at the ætiology one does well to pass in review the various functions and habits of the individuals, in the hope of obtaining a clue. For instance, disease of the liver may be a factor in the development of a skin disease, as may also cardiac disease and renal inadequacy. The nervous system is also of importance, though too much stress may have been laid upon it in the past. It is interesting to note the connection that the nervous system has with the skin, as shown by arsenic poisoning, pigmentation and keratosis with neuritis, etc. But when it is remembered that both are of epiblastic origin, light is at once thrown upon this connection. Susceptibility to certain drugs may be congenital or hereditary. Sudden diarrhœa associated with a drug rash, should not be arrested prematurely, for it may be an attempt of the body to eliminate the drug.

In a case of Xanthelasmoidea, Christie Reid[5] noticed a thrombosed vein in one of the patches. On examining the coagulability of the blood he found it to be very high, and accordingly he prescribed citric acid. The patient was an adult aged 21, and the original plaque was at a spot where a blister had occurred and been pricked. No mast cells were found. The patient recovered, and he thinks that the citric acid had something to do with the favourable result, and recommends as a rational treatment the attempt to lower the coagulability of the blood.

Intolerable pruritus is sometimes associated with alkalinity of the urine. Leo[6] reports such a case and treated the same successfully with mineral acids. In two other cases he has met with the same, one of general pruritus, and the other of pruritus vulvæ. The acid treatment was successful in both cases. Rey[7] suggests the washing out of the colon in children suffering from eczema, as in cases he has treated in this way, even when no obvious disorder of the bowel was present, good resulted. As a proof of a possible nervous origin of psoriasis Balzer and Faure Beaulieu[8] report a case which followed upon the shock of a man seeing one of his children nearly run over. He had never had any form of skin disease before, nor had any member of his family. In association with this one recalls Dore's[9] report of psoriasis following vaccination in three cases. The relationship between cutaneous angiomata and malignant disease has often been discussed. Symmers[10] has studied 400 cases with relation to the age at which they are found. He finds that they are simply a concomitant of the decay of advanced years; and in both young and old are probably significant of some form of well-marked arterial degeneration.

[1] Amer. Jour. Med. Sci., February. [2] Lancet, May 16. [3] Brit. Jour. of Dermatol., June. [4] Brit. Med. Jour., May 16. [5] Lancet, May 30. [6] Brit. Med. Jour., March 7. [7] Brit. Med. Jour., March 7. [8] Amer. Jour. Med. Sci., January 1903. [9] Brit. Jour. of Dermatol., September 1902. [10] Brit. Med. Jour., March 21.

